# A Data-Centric Analysis of the Impact of Non-Electric Data on the Performance of Load Disaggregation Algorithms

**DOI:** 10.3390/s22186914

**Published:** 2022-09-13

**Authors:** João Góis, Lucas Pereira, Nuno Nunes

**Affiliations:** ITI/LARSyS (Lisbon and Funchal), Instituto Superior Técnico (Lisbon), 1049-001 Lisboa, Portugal

**Keywords:** non-electric data, building and consumer characteristics, data-centric analysis, disaggregation performance, non-intrusive load monitoring

## Abstract

Recent research on non-intrusive load monitoring, or load disaggregation, suggests that the performance of algorithms can be affected by factors beyond energy data. In particular, by incorporating non-electric data in load disaggregation analysis, such as building and consumer characteristics, the estimation accuracy of consumption data may be improved. However, this association has rarely been explored in the literature. This work proposes a data-centric methodology for measuring the effect of non-electric characteristics on load disaggregation performance. A real-world dataset is considered for evaluating the proposed methodology, using various appliances and sample rates. The methodology results indicate that the non-electric characteristics may have varying effects on the performances of different building appliances. Therefore, the proposed methodology can be relevant for complementing load disaggregation analysis.

## 1. Introduction

The continuous reduction of greenhouse gas emissions is necessary for a more sustainable global energy distribution. In this regard, the building sector in particular contributes to a significant share of energy emissions [1], and hence the best practices for energy efficiency must be implemented in this area. Many countries have invested considerably over the years to promote the development of smarter electric grids [2]. Smart grids have been developed to produce efficient and effective energy management and control, save energy, and offer a more reliable solution in comparison with conventional power grids [3]. Through the installation of smart metering systems, consumers have access to novel data-based services that enable them to monitor the overall building consumption and user-centric information that goes beyond the traditional billing [4,5].

Smart meters only measure the total building consumption. However, recent research has shown that consumers are more inclined to reduce their consumption if information at the appliance level is provided [6,7]. In this regard, non-intrusive load monitoring (NILM) [8], or load disaggregation, was proposed to estimate the aggregate and appliance (load) electricity consumption from individual meter measurements. The methods in NILM include data acquisition, feature extraction, event detection, load identification, and energy disaggregation, which have been addressed adequately by signal processing and machine learning techniques over time [6].

In terms of load identification and energy estimation, the sample rate of smart meters influences the detection of appliance signatures. The sample rates in NILM datasets typically range from 1 Hz (low frequency) to several kHz (high frequency) [9]. Although the quality of load identification may be improved when high-frequency datasets are considered, the hardware is expensive and requires significant computation time for preprocessing and disaggregation in real-time scenarios [6]. In particular, the recent deep neural network NILM algorithm (DNN-NILM) benefits from high-frequency data and outperforms traditional NILM algorithms [10,11,12]. Nonetheless, NILM algorithms may achieve satisfactory accuracy with data sampled at low frequencies [13]. This is particularly useful because low-frequency datasets are expected to become widely available in the future [14].

Despite the importance of energy data factors in determining the quality of disaggregation, non-electric factors also affect energy consumption in buildings. For instance, building and consumer factors such as the dwelling type, total floor area, dwelling age, number of occupants, and consumer education level affect energy consumption patterns [15,16,17,18]. For instance, a large number of occupants typically results in higher energy consumption. In addition, the energy consumed in apartments is generally lower in comparison with detached dwellings [19,20]. The impact of a building’s characteristics on appliance usage patterns has been analyzed in recent research works. For instance, in [21], a methodology was proposed to formalize the detection of appliance consumption patterns, which was then used for the analysis of consumption profiles.

In order to improve load disaggregation analysis and practices, non-electric characteristics have been suggested to be incorporated into NILM methodologies [22,23,24]. In particular, in [24], an energy efficiency assessment method based on NILM was improved by considering both electric and non-electric data. The performances of NILM algorithms can also be enhanced if the algorithms are optimized for specific dataset characteristics, such as usage patterns and appliance types [25]. According to [26], algorithm optimization is particularly important because the algorithms’ performances tend to deteriorate in new contexts, such as those with usage patterns that are typically different from those the algorithms have been trained on. Since the emergence of usage patterns may be associated with specific non-electric characteristics, it is interesting to investigate the association between the quality of disaggregation and the observed non-electric data.

In this work, a data-centric methodology is proposed for analyzing the overall effect of building and consumer characteristics on the performances of NILM algorithms for individual appliances. This involves estimating regression models for the performance samples of specific building appliances that have previously been obtained for a group of buildings. The regression models are composed of the characteristics that are more statistically significant for describing the performance samples, along with the corresponding effects of each characteristic. The methodology is applied in a realistic scenario for various appliances and at different sample rates. The acquired results demonstrate that the methodology captures the overall effect of each characteristic on the performance samples obtained and, more specifically, that the magnitude of the effects varies depending on the appliance under consideration.

The remainder of this paper is structured as follows. Section 2 presents the background information and related work. Section 3 describes the proposed methods. Section 4 describes an experiment for the methodology’s assessment in a realistic scenario. In Section 5, the experiment’s results are obtained and discussed. Finally, the main conclusions, limitations, and an outlook on further research are provided in Section 6.

## 2. Related Work

In recent years, an increasing amount of research has been conducted to understand and improve the data quality across machine learning disciplines [27]. Similar to other domains, NILM research developments have mostly adopted model-centric approaches due to the continuous progress of machine learning and deep learning algorithms [28]. As a result, several algorithms were proposed and often validated in a small number of datasets. As a consequence of the growing number of collected and generated datasets, many algorithms have poor performance when applied to different datasets [29]. Thus, data-centric approaches for NILM have gained more attention, which enables the extraction of valuable information from the data and improves energy disaggregation analysis.

In the NILM community, there is growing interest concerning the analysis of non-electric data, such as building and consumer characteristics, and how it affects the algorithms’ performances. Indeed, load disaggregation analysis and practices may be improved by including this type of characteristic [22,23,25,26].

In [30], a methodology was proposed for predicting the consumption of each appliance based on the similarity of the building and consumer characteristics. The buildings are grouped according to the similarity of their characteristics following a nearest neighbor’s approach. Then, to predict the consumption in an untested building, an average across similar buildings is calculated. Such a strategy, however, might not be helpful in the case of unusual buildings or appliances. In [31], a feature-based matrix factorization approach was proposed for predicting individual appliance consumption in buildings based on aggregate data and observation of the building and consumer characteristics. In particular, it appears that including building characteristics in the matrix factorization, such as the area and number of occupants, increases disaggregation accuracy. This method has been shown to improve the predictions in comparison with the method in [30].

In [32], a reverse approach was proposed in order to estimate building characteristics using the results of energy disaggregation. The estimation process is directly affected by algorithm selection. Hence, caution is required when making this decision.

From the previous works, there is evidence in favor of the association between building characteristics and disaggregation performance. When analyzing this association, it is interesting to consider datasets that include both consumption data and building information. However, this is not always the case for NILM datasets. In [25], the authors proposed a framework that enables the generation of appliance consumption data by simulating the building and consumer characteristics. Despite the fact that the data are synthetic, the framework allows for the creation of numerous datasets that may be used to assess how certain factors affect the algorithms’ performances.

In this paper, a data-centric approach is proposed in order to directly assess the effect of building and consumer characteristics on disaggregation performance. With respect to [31], the methodology complements the prediction of appliance consumption with the characteristics that have a higher impact on each appliance. In comparison with [30], this methodology aims to provide a more reliable method of evaluating the effect of a building’s characteristics on disaggregation accuracy. Although the framework proposed in [25] enables the generation of synthetic data that might be used for assessing the methodology, real-world data are initially used for evaluation in a more realistic context.

## 3. Methods

In this section, the proposed methodology for assessing the overall effect of non-electric characteristics on the algorithms’ performances is presented.

Assume that *M* disaggregation algorithms are employed for estimating the individual consumption of appliances app=1,⋯,I from the aggregate data, sampled at freq Hz. The disaggregation performance of the *M* algorithms for a specific appliance app across the *J* buildings can be grouped into a matrix ${\left[Y_{j,m,freq}^{app}\right]}_{j=1,m=1}^{J,M}$ of dimensions J×M.

Based on the previous disaggregation experiments, the overall effects of the characteristics on the performance samples are assessed through regression analysis. A regression model is fit to a performance sample ${\left[Y_{j,m,freq}^{app}\right]}_{j=1}^{m}$ for a given appliance app and disaggregation algorithm *m* applied to data sampled at freq Hz:

(1) $$f_{\lambda }\left(Y_{j,m,freq}^{app}\right)=a_{1}X_{1j}+a_{2}X_{2j}+\cdots +a_{k}X_{kj}+b,$$

where $X_{ij}$, i=1,⋯,k are the variables, the categorical variables are modeled as dummies [33,34] (i.e., for each variable, l−1 out of the total levels *l* enter the regression predictor as binary variables, and the effect of each level is evaluated in relation to a level that was left out (reference group)), $a_{i}$ are the regression coefficients, *b* is the intercept to be estimated, abd $f_{\lambda }$ is any needed Box–Cox transformation (BCT) [35] to ensure that the distribution of ${\left[f_{\lambda }\left(Y_{j,m,freq}^{app}\right)\right]}_{j=1}^{J}$ is closer to the normal distribution than ${\left[Y_{m,freq}^{app}\right]}_{j=1}^{J}$, where $f_{\lambda }\left(y\right)=\frac{y^{\lambda }-1}{\lambda }$ for λ∈[−5,5]∖{0}; otherwise $f_{\lambda }\left(y\right)=\log y$ for λ=0.

In order to inspect if the performance samples ${\left[f_{\lambda }\left(Y_{j,m,freq}^{app}\right)\right]}_{j=1}^{J}$ and ${\left[f_{\lambda }\left(Y_{j,m,freq}^{app}\right)\right]}_{j=1}^{J}$ are normally distributed, the Shapiro–Wilk statistical test [36] is considered. According to the test, there is evidence that the sample distribution originates from the normal distribution if the *p*-value is greater than or equal to a threshold of 0.5. For supplementary purposes, the histogram visualization and kernel density estimator (KDE) of the sample distributions are also inspected.

Then, the variables to be included in Equation (1) are selected using the backward stepwise selection (BSS) method [34], which estimates the coefficients $a_{i}$ and *b*. In the BSS, the significance of each variable is determined by the *p*-value of the *T* statistic, which is compared at each step to a reference value that is typically set to 0.01,0.05, or 0.10. If the *p*-value of the *T* statistic is inferior to the reference value, then the effect of the variable is significant for explaining the disaggregation performance. In this work, the reference value was set to 0.10, which relaxed the required statistical significance for a variable to be included in the selected regression model. At each step of the BSS, the variable with the highest *p*-value is excluded until all of the associated *p*-values are less than 0.10, at which point the selected variables are returned. To assess the goodness of fit of the model, the Durbin–Watson test and the coefficient of determination R${}^{2}$ are also examined at each step of the BSS. The former determines whether the model residuals are auto-correlated. In a case where there is no correlation, there is evidence for normality of the residuals. The latter assesses how well the sample data fit the estimated regression model [33,34].

## 4. Experiment Specification

In this section, an experiment is designed in order to illustrate how the proposed methodology can be applied.

### 4.1. Dataset and Non-Electric Characteristics

The REFIT Electrical Load Measurements (REFIT) dataset [37] was considered. This dataset contains cleaned aggregate and individual appliance power consumption data for 20 United Kingdom (UK) residential buildings (9 sub-metered appliances per house) labeled with numbers {1,2,⋯,21}∖{14}. The data were originally timestamped and sampled at 8 s for 2 years (2013–2015). The appliances considered were the washing machine (WM), dishwasher (DW), and microwave (MW) over a year (from June 2014 to June 2015). These appliances were chosen to illustrate the proposed methodology and also because the corresponding consumption depends on the consumer’s usage patterns (human activities and schedules), which are consequently associated with particular building and consumer characteristics [38]. In contrast, cool appliances, such as the refrigerator and freezer, are user-independent, operating continuously in cycles. Therefore, the impact of the building and consumer characteristics on the disaggregation performance should be small. By inspecting the REFIT dataset houses, there were 11 out of the 20 houses that contained at least 1 WM, 1 DW, and 1 MW [37] (i.e., houses 2, 3, 5, 6, 9, 10, 11, 13, 15, 18, and 20), which would be considered throughout the experiment.

The building and consumer characteristics that were available from the metadata were the number of occupants (OC), dwelling size (S), number of appliances (AP), dwelling age (DA) and dwelling type (DT). The term variable is used in this study to denote the characteristics to be considered for the methodology. Observe that variable *S* is given as the number of bedrooms because this is a more common measure of dwelling size in the UK. The variable *DA* indicates the construction period of the dwelling. Additionally, the variable *AP* indicates the total number of electrical appliances in the dwelling. From Table 1, it can be seen that variables *OC*, *S*, and *AP* are integers, with minimum and maximum values of (1,4), (15,49), and (3,4), respectively. In contrast, the variables *DA* and *DT* were categorical, with the most frequent levels being *1965–1974* and *detached*, respectively.

For the variable *DT*, the *detached* *(D)* type refers to stand-alone residential structures without shared outside walls with buildings nearby. The *semi-detached* *(SD)* type is attached to another building by one common party wall, while the *mid-terrace* *(MT)* type is similar to *SD*, but it is situated in the middle of a row of similar houses.

Different data granularity scenarios are taken into account for each appliance to ensure that all appliance activations are recorded over time and the disaggregation performance is not composed of a lack of appliance activations. In this sense, an appliance activation refers to any transition from the off state to one in which the appliance is switched on. For each appliance, the data granularity scenarios are 1/60 Hz and 1/300 Hz for both the WM and DW, respectively, and 1/30 Hz and 1/60 Hz for the MW. In contrast with the MW, smaller data granularity is sufficient to cover all activations in the case of the WM and DW because these appliances are generally activated for longer periods.

### 4.2. Disaggregation Experiments

The algorithms selected for the disaggregation experiments were the DNN-NILM state-of-the-art benchmarks available in NILMTK [10], namely the denoise autoencoder (DAE), sequence-to-point (S2P), and sequence-to-sequence (S2S) algorithms. These algorithms have been shown to outperform the performance of conventional NILM algorithms [11,12]. Despite the fact that high-frequency data yield higher performance for these algorithms, low-frequency data may be adequate to run these algorithms with high accuracy [13,14]. For each algorithm, the data were split between training and testing, with 67% and 33%, respectively, allocated to each. The employed algorithms were trained for 200 epochs with a batch size =512 (i.e., for each of the 200 runs through the training set, the learning algorithm examined 512 equally sized samples of the training set to update the weights). These values were set for illustrative purposes, although the number of epochs was chosen to be greater than in previous works (e.g., 50 *epochs* in [10]) to avoid potential underfitting but also not too large for overfitting. The *batch size* was determined to be between 64 and 1024, which were considered in [10,39], respectively.

### 4.3. Performance Metric and Model Selection

The mean absolute error (MAE) [14,40] was chosen over other metrics because it is based on the L1-norm, which penalizes both small and large errors equally. In contrast, for other metrics such as the root mean squared error (RMSE) [14,40], the performance can be significantly impacted by large errors as opposed to smaller ones because it is based on the L2-norm.

In this experiment, the algorithm that achieved the best performance across the buildings was selected for each case. Three indicators were considered for selecting the algorithm: the frequency of the best performance (lower MAE) across the buildings, the MAE median, and the MAE mean. The mean was chosen as an indicator based on the significance of the two-sample Kolmogorov–Smirnov (TSKS) [41], which tests if pairwise distributions of ${\left[Y_{j,m,freq}^{app}\right]}_{j=1}^{J}$ for m∈{DAE,S2P,S2S} are identical. If the *p*-value for the KS statistic is larger than any of the significance levels of 0.01, 0.05, and 0.10, then there is evidence that the two sample distributions under testing are identical. Since three algorithms were considered, then M=3 and 32=3 TSKS tests were run for each appliance and data granularity. To simplify the notation when the best algorithm was selected, $Y_{j,m,freq}^{app}$ took the place of $Y_{j,freq}^{app}$. In addition, the performance sample was referred to as $Y_{freq}^{app}$.

In order to obtain the effects of the characteristics on the disaggregation performances, the regression models from Equation (1) were then fitted for each appliance and data granularity. The normality of the performance samples was inspected by considering the Shapiro–Wilk test, histogram visualization, and KDE.

### 4.4. Hardware and Software

In terms of software, the experiment was conducted in *Python* 3.6.8. The DNN-NILM algorithms were implemented in the *NILMTK-Contrib* [10] package using the DAE architecture proposed in [39] and the S2S and S2P architectures in [11]. For the estimation of λ for the BCT and to assess the normality of the performance samples $Y_{m,freq}^{app}$ via the Shapiro–Wilk test, the *scipy* package was employed. For model estimation, BSS was carried out using the functionalities of the *statsmodels* package. The computer’s hardware included an Intel i7−8700k CPU, an NVIDIA 1080TI graphics card, and 64 GB of RAM. The code for reproducing the experiments is available at https://anonymous.4open.science/r/Sensors_SI-3AF3/.

## 5. Results and Discussion

In this section, the methodology is applied to the specified experiment for illustration. At first, for each appliance, the disaggregation results are computed for the algorithms considered across a set of buildings. Then, by employing the proposed methodology, the overall effects of the building and consumer characteristics on the disaggregation performance are estimated. Finally, the research findings are discussed.

### 5.1. Appliance-Level Analysis

#### 5.1.1. Washing Machine

The disaggregation performances of the algorithms for the washing machine are displayed in Table 2. For 1/60 Hz, the *S2P* had a lower MAE than the *DAE* and *S2S* for 10 out of 11 houses, as well as a lower MAE median across the houses. By computing the TSKS tests for 1/60 Hz, the *p*-values of the KS statistic were larger than any *p*-value of significance (i.e., $p_{1/60}^{DAE,S2S}=0.83$, $p_{1/60}^{DAE,S2P}=0.48$, and $p_{1/60}^{S2P,S2S}=1$ are all greater than 0.10). Since there was evidence for the pairwise distributions to be identical, it was adequate to use the MAE mean for comparison between the obtained performance samples. *S2P* was also the algorithm with the lower MAE mean. Hence, *S2P* was chosen for disaggregating the washing machine at 1/60 Hz.

Similarly, for 1/300 Hz, *S2P* seemed to attain the best performance across the houses and a lower average MAE. From the TSKS tests for 1/300 Hz, it was adequate to consider the MAE mean across the houses since there was evidence that the algorithms’ performance samples were identical (i.e., $p_{1/300}^{DAE,S2S}=p_{1/300}^{DAE,S2P}=p_{1/300}^{S2P,S2S}=0.83>0.10$). Therefore, *S2P* was the chosen algorithm.

The *S2P* performance samples had small differences across the data scenarios, and the conclusions were similar (Table 2), which indicates that the WM activations were captured for both scenarios. If the data were sampled with lower granularity (e.g., one sample for each 15 min), the disaggregation results could possibly show more differences between the data scenarios.

In order to inspect the overall effects of the characteristics, the *S2P* performance samples $Y_{1/60}^{WM}$ and $Y_{1/300}^{WM}$ (or a transformation of these samples) were fitted to a regression model as in Equation (1). From the Shapiro–Wilk test, there was evidence that the sample distributions were not normally distributed (i.e., $p_{1/60}=0.04<0.5$, and $p_{1/300}=0.02<0.5$). The fitted λ for the BCT was close to zero in both cases (i.e., $\lambda_{1/60}^{WM}\approx 0.10$ and $\lambda_{1/300}^{WM}\approx 0.03$) and were thus approximated to zero (log-transformation) to facilitate further interpretation.

**Figure 1:**
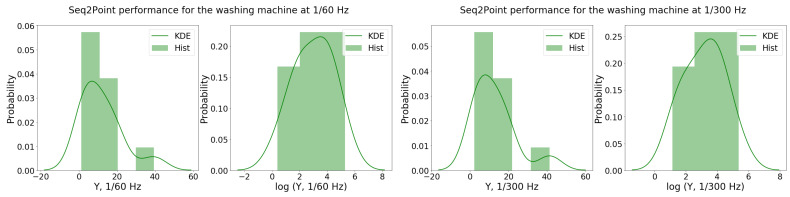
Histogram and KDE of the S2P performance samples for the washing machine for both data scenarios, $Y_{1/60}^{WM}$ (**left**) and $Y_{1/300}^{WM}$ (**right**), and respective log-transformations.

When the log-transform was considered for $Y_{1/60}^{WM}$ and $Y_{1/300}^{WM}$, the *p*-value of the Shapiro–Wilk test improved (i.e., $p_{1/60}^{log}=0.87>0.5$ and $p_{1/300}^{log}=0.86>0.5$). This is also indicated through the histogram visualization and KDE in Figure 1, with the log-distributions of $Y_{1/60}^{WM}$ and $Y_{1/300}^{WM}$ closer to the normal distribution and, in particular, more symmetric.

The selected models are represented in Equation (2)

(2) $$\left\{\begin{array}{c} \log\left(Y_{1/60,j}^{WM}\right)=0.80\times OC_{j}+0.98\\ \log\left(Y_{1/300,j}^{WM}\right)=0.71\times OC_{j}+1.36 \end{array}\right.$$

for j=1,⋯,11 by carrying out BSS across the variables in Table 1. Although the variable *DA* could be relevant for explaining appliance consumption patterns, it was not considered because it comprised a large number of levels (seven in total), each of which was not sufficiently represented due to the small number of houses in the datasets. Furthermore, some DA levels were redundant (e.g., *post 2002* and *2005*), and there was a missing value for house 2. In contrast, for the variable *DT*, the number of levels was smaller, each of which was represented more. The reference group for *DT* was chosen to be *D* (without loss of generality).

By inspecting Equation (2), it seems that the dwelling size, number of appliances, and dwelling type had no significant impact on the disaggregation performance for both data scenarios because these variables were not included in the selected models (estimated coefficients equal to zero). In contrast, the characteristic with a higher impact on disaggregation of the washing machine was the number of occupants for both data scenarios. The average effect of an integer variable on the disaggregation performance sample for a one-unit increase while the remaining variables were fixed was equal to $(e^{a}-1)\times 100\%$, where *a* is the estimated variable coefficient. In a similar fashion, the average impact of the dummies was calculated in relation to the chosen reference group when using the same formula. In the context of the disaggregation problem, if the variable coefficient is negative, then the performance improves, and vice-versa.

Hence, if the number of occupants is increased by one unit, with the remaining variables being fixed, then the disaggregation performance decreases, on average, by a factor of 1.23 for 1/60 Hz (1.03 for 1/300 Hz). For the remaining characteristics, the effects are not relevant.

#### 5.1.2. Dishwasher

The disaggregation results for the DW in Table 3 demonstrate that *S2S* achieved the best performance overall in both data scenarios. For 1/60 Hz, the MAE median and MAE mean for *S2S* were improved in comparison with the other algorithms. Since the distributions of the performance samples appeared to be the same in the TSKS tests, using the mean for comparison was appropriate (i.e., the *p*-values of the KS statistic $p_{1/60}^{DAE,S2S}=0.83$, $p_{1/60}^{DAE,S2P}=1$, and $p_{1/60}^{S2P,S2S}=1$ were all greater than 0.10).

Similarly, for 1/300 Hz, *S2S* attained the best performance across the houses in general and a lower MAE median and MAE mean. Again, the TSKS tests show that it is adequate to use the mean for comparison of the performance samples (i.e., $p_{1/60}^{DAE,S2S}=0.83$, $p_{1/60}^{DAE,S2P}\;=\;0.83$, and $p_{1/60}^{S2P,S2S}=1$ were all greater than 0.10).

In this case, the *S2S* performance samples exhibited some differences across the data scenarios, mainly for houses 2, 6, and 13. Hence, it is possible that a fraction of dishwasher activations at 1/60 Hz were also not detected for 1/300 Hz.

**Figure 2:**
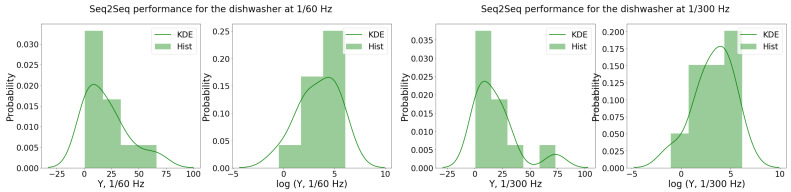
Histogram and KDE of the *S2S* performance samples for the dishwasher for both data scenarios, $Y_{1/60}^{DW}$ (**left**) and $Y_{1/300}^{DW}$ (**right**), and respective log-transformations.

The *S2S* performance samples across the houses, denoted as $Y_{1/60}^{DW}$ and $Y_{1/300}^{DW}$, were fitted to a regression model. From the the Shapiro–Wilk test, the sample distributions did not seem to be normally distributed (i.e., $p_{1/60}=0.05<0.5$ and $p_{1/300}=0.01<0.5$). A BCT is suggested to be carried out with $\lambda_{1/60}^{DW}\approx 0.20$ and $\lambda_{1/300}^{DW}\approx 0.21$. Since these values were close to zero, the log transformation was used to approximate the BCT. Indeed, the *p*-value of the Shapiro–Wilk test improved when the log-transform was considered (i.e., $p_{1/60}^{log}=0.47\approx 0.5$ and $p_{1/300}^{log}=0.64>0.5$). This can also be observed through the histogram visualization and KDE in Figure 2, in which the log-distributions are closer to the normal distribution. The selected regression models are represented in Equation (3) using BSS:

(3) $$\left\{\begin{array}{l} \log\left(Y_{1/60,j}^{DW}\right)=1.37\times OC_{j}-0.11\times AP_{j}-2.80\times DT\_SD_{j}+3.71\;\\ \log\left(Y_{1/300,j}^{DW}\right)=1.36\times OC_{j}-1.68\times DT\_SD_{j}+0.12 \end{array}\right.$$

According to Equation (3), the dwelling size had no relevant effect on the performance for both scenarios. For 1/60 Hz, a one-unit increase in the number of occupants, with the remaining variables held constant, reduced the performance by a factor of 2.94 (similar to 1/300 Hz) on average. Furthermore, if the dwelling was semi-detached, this improved the performance by 94% (81% for 1/300 Hz) when compared with a detached dwelling type. The number of appliances did not seem to be significant for explaining the performance because the effects of this variable varied depending on the data scenario considered. For 1/60 Hz, a one-unit increase in the number of appliances improved the performance by 10% on average, although this variable had no significance for 1/300 Hz.

#### 5.1.3. Microwave

The disaggregation results for the MW in Table 4 show that *S2P* performed the best overall for either the 1/30-Hz or 1/60-Hz granularities. For 1/30 Hz, the MAE median and MAE mean were lower for *S2P*. The mean was adequate to be considered for comparison, as the TSKS tests indicated that the performance samples followed similar distributions (i.e., the *p*-values of the KS statistic $p_{1/60}^{DAE,S2S}=p_{1/60}^{DAE,S2P}=p_{1/60}^{S2P,S2S}=1$ were all greater than 0.10).

For 1/60 Hz, *S2P* attained the best performance for most houses and also had a lower MAE median and average MAE. Again, the TSKS tests indicated that the performance samples followed similar distributions (i.e., $p_{1/60}^{DAE,S2S}=p_{1/60}^{DAE,S2P}=0.48$, and $p_{1/60}^{S2P,S2S}=1$ were all greater than 0.10). Similar to the washing machine, there were no significant differences in the *S2P* performance samples across the two data scenarios).

**Figure 3:**
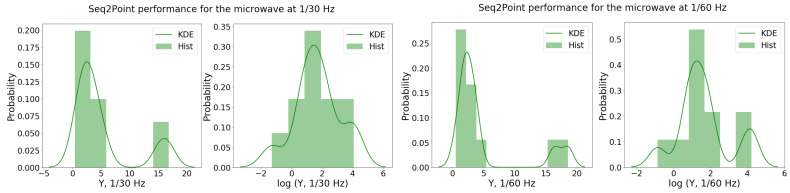
Histogram and KDE of the *S2P* performance samples for the microwave for both data scenarios, $Y_{1/30}^{MW}$ (**left**) and $Y_{1/60}^{MW}$ (**right**), and respective log-transformations.

The *S2P* performance samples $Y_{1/30}^{MW}$ and $Y_{1/60}^{MW}$ were then fitted to a regression model. From the Shapiro–Wilk tests, the sample distributions did not seem to come from a normal distribution (i.e., $p_{1/30}\approx 0<0.5$ and $p_{1/60}\approx 0<0.5$). A BCT was suggested with $\lambda_{1/30}^{MW}\approx 0.03$ and $\lambda_{1/60}^{MW}\approx -0.19$. Since these values were relatively close to zero, the log transformation was considered. Indeed, the *p*-value of the Shapiro–Wilk test for the log-distributions of $Y_{1/30}^{MW}$ and $Y_{1/60}^{MW}$ improved (i.e., $p_{1/30}^{log}=0.58>0.5$ and $p_{1/60}^{log}\approx 0.21<0.5$). This is also indicated by inspection of the histograms and KDE, as shown in Figure 3.

The selected models’ interpretations were similar for both data scenarios, as observed in Equation (4):

(4) $$\left\{\begin{array}{l} \log\left(Y_{1/30,j}^{MW}\right)=2.29\times S_{j}-5.81\;\\ \log\left(Y_{1/60,j}^{MW}\right)=2.41\times S_{j}-6.26 \end{array}\right.$$

It seems that the number of occupants, the number of appliances, and the dwelling type had no significant effect on the disaggregation performance. In contrast, the dwelling size was the characteristic with a higher impact. By increasing the number of bedrooms in one unit, the disaggregation performance decreased on average by a factor of 8.87 for 1/30 Hz (10.13 for 1/60 Hz).

### 5.2. Discussion

In this subsection, the results obtained in the previous experiment are discussed. As can be seen in Table 5, the building and consumer characteristics had varying effects on the disaggregation performance, depending on the appliance analyzed and considering the algorithm that achieved the best overall performance across the buildings.

For the WM models, the number of occupants was the only relevant characteristic for explaining the disaggregation performance (Table 5). Because the effect was positive, a large number of occupants decreased the disaggregation performance on average. According to [15], a higher energy consumption is expected when the number of occupants is large. In this experiment, the average number of WM activations (at 1/60 Hz) across the houses with 2 occupants was equal to 69.6, whereas the number of activations was equal to 118.4 for houses with 4 occupants, which is obviously higher. When there are more appliance activations, the learning ability of the algorithms may be reduced because there are more usage patterns to correctly identify. This is consistent with the positive effect for the number of occupants.

Concerning the DW models, the conclusions were slightly different for the two data scenarios. The number of appliances was included in the regression model for 1/60 Hz. However, the number of appliances did not seem to be completely relevant for explaining the performance, as its significance varied depending on the data granularity. The 9 sub-metered appliances accounted for an average of 41% of the total energy consumed across the houses, while the remaining appliances only accounted for a minor fraction of the total energy. The latter appliances were either not used regularly or for long periods of time, and thus their share in the house consumption was more meaningful for larger data granularities, such as 1/60 Hz, than for smaller granularities, where their consumption was not relevant. The number of occupants and dwelling type (semi-detached) seem to be relevant characteristics for both data scenarios (Table 5). Because the effect of the number of occupants is positive, a large number of occupants decreases the disaggregation performance, with a similar interpretation as that for the WM. If the dwelling type is semi-detached, then the disaggregation performance is expected to improve in comparison with detached dwellings (negative effect). By inspecting the houses used in the experiment, the amount of energy consumed by DWs in semi-detached houses was roughly equal to 3% during the test period, while for detached houses, it was roughly equal to 1.6%. This finding suggests that DW consumption is higher for semi-detached houses in comparison with detached houses and consequently may improve the learning ability of the disaggregation algorithm. Nevertheless, the dwelling type can be correlated with other indicators, such as house income, which were not recorded in the dataset but may help to interpret the results.

Concerning the MW models, the dwelling size was the only relevant characteristic for explaining the disaggregation performance, having a positive effect (Table 5). With each additional bedroom, the disaggregation performance decreased on average. By inspecting the houses in the test period, the average number of MW activations was roughly equal to 140 for houses with 3 bedrooms, while for houses with 4 bedrooms, it was roughly equal to 282. Similar to the WM, additional WM activations may compromise the learning ability of algorithms because more usage patterns would need to be correctly identified.

The number of appliances seemed to be an irrelevant characteristic for all appliances, which seems to be consistent with the fact that nine appliances that were sub-metered for all buildings accounted for a sizable portion of the total energy consumed. Additionally, according to [17], semi-detached dwellings tend to be more energy efficient than detached dwellings. The validity of this statement could be further investigated in the case of DWs. Although the dwelling size was a relevant characteristic for MWs, it was not relevant for WMs or DWs, which seems to be consistent with the fact that large buildings are not necessarily fully occupied [17], compromising potential associations. This effect should be further investigated.

Concerning WMs and MWs, the findings were very similar for both data scenarios. However, the sample rate can be a determining factor in the results and is worth investigating, as was observed for the DWs. Additionally, the DW models were more complex than the WM and MW models because more variables were selected for explaining the performance.

Therefore, the experiments carried out in Section 5.1 demonstrate that the methodology allows for the identification of the non-electric characteristics that are more statistically significant for explaining the obtained disaggregation performances and estimating their respective effects. The estimated effects may vary depending on the appliance and data scenario considered. The methodology was also shown to be relatively simple, flexible, and generalizable to a larger number of buildings. In fact, the estimated effects of the characteristics for each appliance are expected to be more accurate if a larger set of buildings is considered.

## 6. Conclusions

The methodology presented in this work provides a flexible and understandable analysis of the overall effects of non-electric characteristics on the performance of NILM algorithms for specific appliances. The REFIT dataset was used to illustrate the methodology in a realistic scenario. The overall impact of the non-electric characteristics on the performance of algorithms was analyzed for various appliances across a set of buildings at different sample rates. The experiment’s results led to the conclusion that the magnitude of the effects of the non-electric characteristics vary depending on the appliance analyzed. In addition, the magnitude of the effects can be affected in case the selected sample rates impact the detection of appliance usage. Therefore, the methodology can be used to complement the NILM analysis with data that indirectly impacts the consumption data. In the next subsections, the implications and potential applications of this research work are addressed, as well as its limitations and further research.

### 6.1. Research Implications and Potential Applications

The experimental results demonstrate that the effects of each characteristic on the algorithms’ performances varied depending on the appliance analyzed, which may directly affect consumption estimation and usage pattern and anomaly detection. Therefore, a research implication of this work would be to incorporate the methodology into NILM analysis. With respect to [25], the disaggregation algorithms could be optimized for the characteristics with a more noticeable impact on the performance. A potential application would be to provide a more detailed analysis of the overall energy efficiency in a building, as well as the creation of energy communities.

Moreover, based on the estimated regression models, the proposed methodology may be used to predict the disaggregation performances for the individual appliances. The performance could be estimated in untested buildings if the non-electric characteristics were observed. For buildings with similar characteristics, the regression models estimated similar disaggregation performances.

Furthermore, this methodology can also motivate further research on the topic of load disaggregation complexity in terms of the non-electric characteristics that go beyond electric signals. With respect to [12,42], the proposed methodology can complement the results of complexity measurements based on appliance states and time-series similarity, with more information on other characteristics that constitute the building environment.

### 6.2. Limitations and Future Work

Although the methodology presented in this work can improve NILM analysis, there are some limitations regarding the experiment’s specifications and methods that should be taken into consideration.

In terms of the experiment’s specifications, the selected algorithms for each case were limited to *M* algorithms. Despite the employed DNN-NILM algorithms being state-of-the-art and widely used for benchmarking disaggregation performance, it would be adequate to use more algorithms for comparison. For instance, *WindowGRU* [43] is also a DNN-NILM algorithm that could be included. Furthermore, although the MAE is a standard metric for evaluating disaggregation performance in energy estimation, it was not normalized, which affected the comparison among NILM approaches. Therefore, it would be adequate to use a normalized metric to supplement the interpretation of the results, such as the normalized RMSE [14] or normalized disaggregation error (NDE) [40].

Furthermore, the sample rates for each appliance should be chosen accordingly. Otherwise, the effects of some characteristics can vary significantly across data scenarios and, in certain cases, be misleading. For each appliance, it would also be interesting to consider additional suitable sample rates and examine how much the variables’ effects vary, such as using lower sample rates for washing machines and dishwashers in comparison with the microwave, because the former appliances generally have longer activation times.

This methodology also depends on the availability of non-electric data. However, due to privacy concerns, this type of data is frequently unavailable in datasets. In this work, four building and consumer characteristics were considered in the analysis. However, a number of characteristics that possibly impact energy consumption patterns were not included in the dataset, such as consumer education level and income [15,17]. Although data privacy is an important concern related to the collection of these types of data, this work can motivate the creation of more energy datasets that either keep data privacy or record non-electric data.

The selected regression models for each appliance are also influenced by the number of buildings recorded in the dataset. In this work, a total of 11 buildings—a relatively small sample—was taken into account. The dwelling age was not considered because the variable levels were not sufficiently represented. The effects of the variables in the selected models are more accurate when larger samples of buildings are considered. For a greater generalization, the proposed methodology could be applied to datasets with larger numbers of buildings, such as the Fresh Energy [44] and HES [45] datasets with 200 and 250 buildings, respectively. Another option would be to consider synthetic data for methodology evaluation, which could be generated based on the simulation procedure in [25].

Finally, the selection of models for each appliance depends on the reference *p*-value chosen for the BSS, which impacts the effects of the characteristics. The reference *p*-value was chosen to be 0.10, but other significance levels could be considered, such as 0.05 and 0.01. Finally, the inclusion of nonlinear functions of variables in the regression model could be tested, such as polynomials [33], and they might be evaluated to analyze whether the effects of characteristics are nonlinear.

## Figures and Tables

**Table 1:** Building and consumer characteristics for the REFIT dataset houses considered in the experiment.

Houses											
	**2**	**3**	**5**	**6**	**9**	**10**	**11**	**13**	**15**	**18**	**20**
OC	4	2	4	2	2	4	1	4	1	2	2
S	3	3	4	4	3	3	3	4	3	3	3
AP	15	27	44	49	24	31	25	28	19	34	39
DA	-	1988	1878	2005	1919–1944	1919–1944	1945–1964	post 2002	1965–1974	1965–1974	1965–1974
DT	SD	D	MT	D	D	D	D	D	SD	D	D

**Table 2:** Disaggregation performances of *DAE*, *S2P*, and *S2S* for the washing machine at 1/60-Hz and 1/300-Hz data granularities. The indicators considered for selecting the best algorithm (orange) were the FBP, MAE median, and MAE mean.

MAE (1/60 Hz)												Indicators		
**Houses**												**FBP**	**Median**	**Mean**
	2	3	5	6	9	10	11	13	15	18	20			
DAE	32.30	27.01	46.16	5.49	15.58	30.69	10.21	16.69	5.65	3.27	7.74	0	15.58	18.25
S2P	15.62	19.98	39.37	3.07	11.63	19.26	4.85	10.21	3.33	1.32	4.85	10	10.21	12.14
S2S	18.94	19.87	42.71	3.97	12.56	21.66	4.90	11.21	3.89	1.40	6.39	1	11.21	13.41
**MAE (1/300 Hz)**														
DAE	20.46	30.74	44.40	6.23	14.68	26.34	7.97	17.21	5.95	3.29	7.17	0	14.68	16.77
S2P	16.55	19.72	41.39	2.96	12.01	18.73	6.80	10.72	3.60	2.21	6.26	7	10.72	12.81
S2S	19.99	20.05	44.00	3.76	11.09	20.01	6.75	9.63	3.92	2.34	5.20	4	9.63	13.34

**Table 3:** Disaggregation performances of *DAE*, *S2P*, and *S2S* for the dishwasher at 1/60-Hz and 1/300-Hz data granularities. The algorithm that attains the best overall performance is selected (orange).

MAE (1/60 Hz)												Indicators		
**Houses**												**FBP**	**Median**	**Mean**
	2	3	5	6	9	10	11	13	15	18	20			
DAE	43.51	28.29	27.23	4.51	18.83	31.11	6.69	62.11	1.27	4.77	6.05	2	18.83	21.31
S2P	53.99	21.26	26.86	3.70	17.93	28.17	5.60	72.66	0.50	4.80	3.94	5	17.93	21.76
S2S	45.07	24.07	23.20	3.61	16.82	30.21	5.72	66.44	0.72	4.15	4.27	4	16.82	20.39
**MAE (1/300 Hz)**														
DAE	28.34	28.25	27.90	5.10	17.49	33.89	5.61	77.68	0.58	5.21	3.74	1	17.49	21.25
S2P	26.95	20.32	20.41	5.42	12.34	33.20	4.24	77.33	0.92	3.80	3.94	3	12.34	18.99
S2S	26.41	20.58	22.12	8.32	12.28	31.72	3.68	73.21	0.49	4.58	3.17	7	12.28	18.78

**Table 4:** Disaggregation performances of *DAE*, *S2P*, and *S2S* for the microwave at 1/30-Hz and 1/60-Hz data granularities. The algorithm that attains the best overall performance is selected (orange).

MAE (1/30 Hz)												Indicators		
**Houses**												**FBP**	**Median**	**Mean**
	2	3	5	6	9	10	11	13	15	18	20			
DAE	3.50	4.06	21.27	4.38	1.98	2.15	1.61	17.46	0.60	2.11	7.04	0	3.50	6.01
S2P	2.86	3.72	16.85	4.21	1.73	1.65	2.83	15.27	0.41	1.91	5.53	5	2.86	5.18
S2S	3.06	3.67	18.28	4.18	1.66	1.65	1.46	16.00	0.48	2.07	5.07	5	3.06	5.23
**MAE (1/60 Hz)**														
DAE	4.24	4.93	21.82	5.06	2.02	2.61	1.57	15.96	0.63	2.35	5.16	1	4.24	6.03
S2P	2.95	3.89	16.39	3.74	1.89	1.57	2.82	18.56	0.55	2.01	2.07	6	2.82	5.13
S2S	3.62	3.63	18.05	4.14	1.58	1.61	1.42	17.74	0.53	2.03	3.90	4	3.62	5.30

**Table 5:** Summary of the building and consumer characteristics with statistically significant and irrelevant effects for the appliances considered in the experiment. Relevant positive effects are indicated by “+”, while negative effects are indicated by “−”.

Appliance	Significant Effects	Irrelevant Effects
Washing Machine	*OC*(+)	*S*,*DT*, *APP*
Dishwasher	*OC*(+), DT_SD(−)	*S*, *APP*
Microwave	*S*(+)	*OC, APP, DT*

## Data Availability

Not applicable.

## Abbreviations

The following abbreviations are used in this manuscript:

AP	Number of appliances
BCT	Box–Cox transformation
BSS	Backward stepwise selection
D	Detached
DA	Dwelling age
DAE	Denoise autoencoder
DNN-NILM	Deep neural network NILM algorithms
DT	Dwelling type
DW	Dishwasher
KDE	Kernel density estimator
MAE	Mean absolute error
MT	Mid-terrace
MW	Microwave
NDE	Normalized disaggregation error
NILM	Non-intrusive load monitoring
OC	Number of occupants
RMSE	Root mean squared error
S	Dwelling size
S2P	Sequence-to-point
S2S	Sequence-to-sequence
SD	Semi-detached
TSKS	Two-sample Kolmogorov–Smirnov
UK	United Kingdom
WM	Washing machine
